# Inter-Country Doctor-to-Doctor Telemedicine Conferences/Consultations Following COVID-19: A Survey of the National University Hospital Council of Japan

**DOI:** 10.1089/tmr.2025.0015

**Published:** 2025-05-20

**Authors:** Kuriko Kudo, Yukiko Hisada, Shintaro Ueda, Makoto Kikukawa, Naoki Nakashima, Tomohiko Moriyama

**Affiliations:** ^1^ International Medical Department, Kyushu University Hospital, Fukuoka, Japan.; ^2^ Department of Medical Education, Faculty of Medical Sciences, Kyushu University, Fukuoka, Japan.

**Keywords:** doctor-to-doctor, internationalization, national survey, Japan, telemedicine, COVID-19

## Abstract

**Background:**

The coronavirus disease 2019 (COVID-19) pandemic has led to the worldwide development of information and communications technology and the widespread use of domestic telemedicine, but the activities and needs of international telemedicine conferences/consultations remain unclear. We examined the experiences, needs, resources, and barriers related to international doctor-to-doctor telemedicine following the COVID-19 pandemic in Japanese national university hospitals.

**Methods:**

In November 2021, a questionnaire was sent to 163 Internationalization Project Team representatives at 43 Japanese national university hospitals. Eighty-two of the representatives were medical staff in charge of internationalization (MI), and 81 were technical staff responsible for telecommunications (TT).

**Results:**

The response rate was 94.2% (MI: 42/43 institutions; TT: 39/43 institutions). Fourteen institutions had been conducting international telemedicine programs with 62 countries. Public health was the most frequently cited topic, followed by nursing, surgery, pediatrics, and gastroenterology. All TT indicated that their institution had installed videoconferencing systems. Nineteen institutions indicated a need for international programs. The most serious barrier was the lack of “human resources” (84%), and this was noted more often by members in institutions without activity (*p* = 0.03). However, there was no difference between groups with and without a support department (*p* = 0.24) or MIs/TTs (*p* = 0.05).

**Discussion:**

The technical barriers for international telemedicine were low after the pandemic. However, there are insufficient human resources to meet the growing needs. In addition to the international coordinators and administrative staff to support smooth communication with overseas partners, there is also a need for personnel to promote activities on their own.

## Introduction 

Medical disparities are one of the major challenges worldwide, and various causes, such as economic conditions, political structure, race and ethnicity, gender, educational background and occupation, and social class, have been analyzed.^1–10^ Differences in the skills of health care professionals and medical training programs are another factor contributing to global health care disparities, and efforts have been made to improve medical training in a variety of health care fields to reduce these disparities.^11–13^ In the field of telemedicine, information and communications technology (ICT) has been used to connect medical professionals and institutions, promoting collaboration with specialists in areas such as physician consultation and conferences, remote diagnosis of pathology (telepathology), and telesurgery, thereby contributing to the reduction of international medical disparities.^14–20^ In a literature review published in 2012, Saliba et al. reported that both doctor-to-doctor and doctor-to-patient programs have been used in international telemedicine in the past 20 years in a variety of areas, including telepathology, telesurgery, emergency, and trauma; most of them were collaborations between high- and low- or middle-income countries.^21^

The Japanese Ministry of Internal Affairs and Communications reports that doctor-to-doctor telemedicine is divided into four categories: “diagnosis support,” “medical treatment support,” “information sharing,” and “education and learning.”^22^ In Japan, the rapid aging of the society and declining birth rate have led to a situation with a maldistribution of doctors and medical resources in the health care system; at the same time, the number of people finding it difficult to go outside due to physical reasons is increasing. In this context, doctor-to-doctor telemedicine is expected to help ensure medical care in local communities, develop efficient and effective medical care provision systems, and improve the efficient working practices of medical professionals through the use of ICT. In addition, due to the recent difficulty in securing doctors and nurses, there is a problem in securing medical services, especially in the fields of emergency, pediatrics, perinatal, disaster, and mental health care, even in urban areas. Furthermore, each region is required to deal with new public health issues, such as measures against lifestyle diseases. The most common scope of service was prefectural, followed by “regions that span multiple secondary medical areas” and “single secondary medical areas.” However, there is no mention of international telemedicine in this report.

International doctor-to-doctor telemedicine is often used in the context of international support. In 2010, the World Health Organization presented its first guidelines for telemedicine, which raised the need for international telemedicine initiatives and the challenge of the lack of models that are tailored to the actual situation.^23^ Guidelines for the use of telemedicine during the coronavirus disease 2019 (COVID-19) pandemic were published in 2022.^24^ They state that when implementing telemedicine programs between two or more countries, it is necessary to follow the telemedicine guidelines of each country or state. In Japan, the Ministry of Foreign Affairs issued the Global Health Diplomacy Strategy, officially positioning health as a pillar of government’s foreign diplomacy.^25^ Japanese official development assistance (ODA) is based on the Development Cooperation Charter, and the Ministry of Foreign Affairs leads the decision-making and implementation process while also reflecting the opinions of other ministries, such as the Japan International Cooperation Agency (JICA). This charter focuses on Asia and expresses the basic principle of supporting economic growth and social development in developing countries through ODA.^26^ Reports on global ODA in the context of COVID-19 also show that ODA in the health and medical fields increased after the pandemic.^27^ In the 2022 JICA Global Health and Medical Care Initiative Interim Review Report, cooperation using telemedicine technology was being implemented in 12 countries in Asia, Central and South America, etc., to support safer and more effective patient treatment in the COVID-19 context.^28^ Here, local medical staff are connected with Japanese specialists, and support is provided for the maintenance of medical equipment and materials, as well as online training and advice.

International doctor-to-doctor telemedicine has also been mentioned in the context of education, albeit infrequently. The benefits of building cross-border medical support systems and online educational activities have been reported.^29,30^ From a technical point of view, these activities are similar to Collaborative Online International Learning in higher education,^31–33^ but hospitals have completely different staff and technical environments than higher education.

This study focused on doctor-to-doctor conferences/consultations as a form of remote medical treatment support and educational activities in various medical fields across international borders. These activities have long been used with general teleconferencing systems, and after the COVID-19 pandemic, there is a possibility that these activities have spread due to the expansion and dissemination of digital health markets.^34–43^ In COVID-19 emergency medicine, more than 500 joint remote rounds were conducted by an interdisciplinary team of medical experts between Uzbekistan and Germany for nearly 200 patients.^44^ The United States provided medical assistance to Sierra Leone in Africa, and remote instruction in intensive care unit operations was provided to physicians and nurses on the front lines of acute response to COVID-19.^19^ However, most of the reports on international telemedicine were from the respective facilities and communities, and national reviews were limited.^45^ Therefore, it was not clear how inter-country doctor-to-doctor telemedicine conferences/consultations are used in a country or region following the COVID-19 pandemic, what the existing needs are, and what kinds of environmental improvements are needed.

In Japan, the National University Hospital Council of Japan established an Internationalization Project Team (I-PT).^46^ One of its aims is to strengthen the exchange of medical professionals among overseas medical institutions utilizing ICT and to build a world-leading remote medical collaborative network. To this end, all 43 of Japan’s national university hospitals assigned staff to two roles: (1) medical staff in charge of internationalization (MI) and (2) technical staff responsible for telecommunication (TT).

This organization operates as a self-help initiative by each university. While compensation for international doctor-to-doctor conference/consultation activities is determined by each university, many of these activities are voluntary. Competitive funding from ODA-related organizations can support these activities, making it possible to secure the necessary budget if the funds are acquired. Moreover, it is thought that such activities can be recognized as achievements in international exchange and education at the national university hospitals where the responsible individuals work. A survey conducted prior to the start of the COVID-19 pandemic found that inter-country doctor-to-doctor telemedicine conferences/consultations were limited by staff shortages, lack of knowledge of where to start, and technical and English communication problems.^45^ However, the COVID-19 pandemic significantly promoted the development and expansion of ICT worldwide,^47–49^ leading to improvements in the technical situation. Therefore, in this study, we surveyed I-PTs again and analyzed the activities, needs, and barriers to inter-country doctor-to-doctor telemedicine conferences/consultations following the COVID-19 pandemic. The research questions addressed in this study are as follows.

- RQ1. What are the activities and needs for inter-country doctor-to-doctor telemedicine conferences/consultations at Japanese national university hospitals after the COVID-19 pandemic?
- RQ2. What level of organizational and technical resources are in place to support inter-country doctor-to-doctor telemedicine conferences/consultations at Japanese national university hospitals?
- RQ3. What are the barriers to promoting inter-country doctor-to-doctor telemedicine conferences/consultations at Japanese national university hospitals? What impact do the activities and support systems in place at facilities, job categories, etc., have on international programs?

## Materials and Methods

### Recruitment

This survey was conducted on the I-PTs, which were established to promote internationalization at national university hospitals in Japan, in order to investigate the international doctor-to-doctor telemedicine activities and needs at national university hospitals in Japan. The inclusion criteria were one MI and one TT appointed to all Japanese national university hospitals, and the exclusion criteria were cases having multiple responses with the same name and cases with incomplete data. In November 2021, a questionnaire was sent by e-mail to 163 I-PT representatives at 43 Japanese national university hospitals, comprising 82 MIs and 81 TTs. Because it is assumed that the information understood by the MI and TT roles will differ, we asked the MIs and TTs of each institution to submit at least one completed questionnaire. The questionnaire, which included demographic features, technical resources, activities, needs, and barriers, was created using Google Forms (Google LLC, Mountain View, CA). For technical resources, only the TTs were asked to respond. The questionnaire for MIs consisted of 34 questions, whereas that for TTs comprised 49 questions. The questions are listed in Table 1.

**Table 1.** Questionnaire Items^a–d^

Category	Questions	Target
Demographic features	1. Please select the one that best describes your job category: *Faculty professor (MD*^c^*)/faculty professor (not MD)/MD (not faculty professor)/administrative staff/researcher/IT*^d^*staff/others* 2. Percentage of time dedicated to telemedicine: *0–20%/21–40%/41–60%/61–80%/81–100%*	MI^a^, TT^b^
Resources	1. Is there any department at your institution that can support international doctor-to-doctor telemedicine conferences/consultations? *-Yes/no* 2. Please select the availability of equipment at your institution for telemedicine activities. A conference room equipped with videoconferencing systems H.323 teleconferencing system (Polycom, Tandberg, Sony, etc.) License for Zoom License for Webex License for Microsoft Teams Other teleconferencing system/streaming system *-Yes/no* 3. For each of the following, please select the level of experience you possess: 3-1. Participation in online meetings (personal terminal) 3-2. Technical staff in online meetings (small conference room) 3-3. Technical staff organizing online meetings 3-4. Operating audio-visual equipment (microphones, projectors, etc.) 3-5. In charge of streaming via webinars, YouTube, etc. 3-6. Troubleshooting in online meetings 3-7. Setting up network devices (routers, switches, etc.) 3-8. Communicating in English *-Well experienced/experienced/neither/less experienced/unexperienced*	MI, TT TT
Activities and needs	1. Are there any issues at your institution that require sharing information or consultation with overseas hospitals? *-Yes/no/not sure* 1-1. (If yes) Is there any need for developing new international doctor-to-doctor telemedicine conference/consultation program(s) at your institution? *-Yes/no/not sure* 2. Have you conducted international doctor-to-doctor telemedicine conference/consultation programs at your institution since the start of the COVID-19 pandemic? *-Yes/no/not sure* 3. (If 2 is yes) Please select the format of the program. 3-1. Lecture/seminar 3-2. Case consultation 3-3. Live demonstration 3-4. Other *-Supported by a commercial company/not supported by a commercial company* 4. (If 2 is yes) Which medical field (department) does the program relate to? *-Free text* 5. (If 2 is yes) Please select the country(ies) that participated in the program:*-Australia, Bhutan, Canada, China, France, Germany, India, Indonesia, Italy, Korea, Malaysia, Myanmar, Nigeria, Philippines, Russia, Spain, Sweden, Switzerland, Thailand, United Kingdom, United States, Vietnam, Other (free text)* 6. Are there any activities at your institution that require sharing information or consultation with domestic hospitals? *-Yes/no/not sure* 6-1. (If yes) Is there any need for developing new domestic doctor-to-doctor telemedicine conference/consultation program(s) at your institution? *-Yes/no/not sure* 7. Have you conducted domestic telemedicine conference/consultation programs at your institution since the start of the COVID-19 pandemic? *-Yes/no/not sure* 7-1. (If 7 is yes) Please select the format of the program. Lecture/seminar Case consultation Live demonstration Other *-Supported by a commercial company/not supported by a commercial company*	MI, TT
Barriers	1. Do you think there are any barriers to the promotion of international doctor-to-doctor telemedicine conference/consultation programs in your institution? *-Yes/no* 1-1. (If yes) Among the items, what is the most serious barrier? *-Shortage of medical staff/shortage of technical staff/shortage of international coordinators or secretaries/shortage of staff in charge at the partner facility/equipment and network issues/shortage of well-equipped meeting rooms/restrictions regarding security policy/others (free text)*	MI, TT

^a^ Medical staff in charge of internationalization.

^b^ Technical staff responsible for telecommunication.

^c^ Medical doctor.

^d^ Information technology.

COVID-19, coronavirus disease 2019.

Questions on TT skills have been updated since the previous survey because videoconferencing (VC) systems have become more popular since the COVID-19 pandemic. In the previous survey, there was an item for “operation of VC system,” but since COVID-19, there were more cases of individuals operating the VC system from their own computers to participate in the programs, and that item was replaced by the following items: “participation in online meetings (personal terminal),” “technical staff in online meetings (small conference room),” and “technical staff in organizing online meetings,” with the questions being asked in a step-wise manner. Additionally, questions regarding streaming and experience supporting online meetings/workshops were added. For activities and needs, information on both domestic and international events was sought for comparison. Regarding needs, those who answered “yes” to the question “Are there any issues at your institution that require sharing information or consultation with overseas hospitals?” were also asked if there was a need for a telemedicine conference/consultation program. Those who answered “no” to the same question were recorded as having “no or not sure” needs. Partner countries for the international telemedicine conference/consultation program were selected from developing and developed countries considered to have active human exchange in the medical field with Japan based on existing data. Hence, 12 developed countries and 10 developing countries were selected, and the remaining countries were left open-ended.^50^ Developed countries were selected from the top 10 countries (the United States, Canada, Germany, the United Kingdom, France, Sweden, Australia, Italy, Switzerland, and Spain) listed in the results of a survey on destinations for doctors studying abroad in an article on M3.com,^51^ a social networking site for Japanese physicians. We also added South Korea and Russia, as they were Japan’s neighboring countries. We selected 10 developing countries (Bhutan, China, India, Indonesia, Malaysia, Myanmar, Nigeria, the Philippines, Thailand, and Vietnam) from a project supported by the National Center for Global Health and Medicine in Japan.^52^

In the case of different perceptions among personnel at the same medical facility, we recorded responses for each facility in addition to the responses for each respondent for activities and barriers.

### Statistical analysis

We used JMP® Pro 16.0.0 (Cary, NC), Microsoft Excel 2019 (Redmond, WA), and IBM® SPSS® Statistics 28.0.1.0 (Chicago, IL) for statistical analyses. Fisher’s exact test was used to compare the number of activities supported by commercial companies and the barriers between groups, at a significance level of 5%.

### Ethical considerations

The Ethics Committee of Kyushu University Hospital (No. 23211-00) reviewed and approved the study protocol. Informed consent was obtained by an opt-out process.

## Results

### Demographic features

A total of 86 staff members (47 MI and 39 TT) responded to the survey, with a response rate of 94.2% (MI: 42/43 institutions; TT: 39/43 institutions). The majority of the MI participants (89%, 42/47) were faculty professors and medical doctors (MD). In contrast, TTs comprised a variety of professionals, such as faculty professors (not MD; 38%, 15/39), administrative staff (26%, 10/39), faculty professors (MD; 23%, 9/39), and information technology staff (13%, 5/39). Almost all participants (MI: 98%, TT: 95%) responded that they dedicated less than 20% of their time to telemedicine (Table 2).

**Table 2.** Characteristics of the Respondents^a–d^

Characteristics	MI^a^ (*N* = 47)	TT^b^ (*N* = 39)
Occupation		
Faculty professor (MD^c^)	42 (89%)	9 (23%)
Faculty professor (not MD)	3 (6%)	15 (38%)
Administrative staff	2 (4%)	10 (26%)
IT^d^ staff	0	5 (13%)
Percentage of effort dedicated to telemedicine		
0–20%	46 (98%)	37 (95%)
21–40%	0	1 (3%)
41–60%	1 (2%)	0
61–80%	0	0
81–100%	0	1 (3%)

^a^ Medical staff in charge of internationalization.

^b^ Technical staff responsible for telecommunication.

^c^ Medical doctor.

^d^ Information technology.

### Resources

**FIG. 1.**
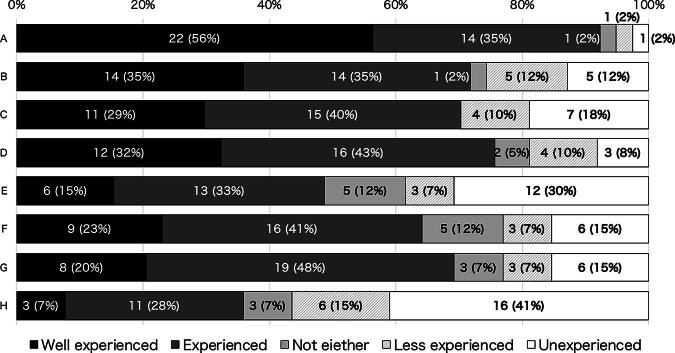
Skills for technicians. **(A)** Participation in online meetings (personal terminal); **(B)** technical staff in online meetings (small conference room); **(C)** technical staff in organizing online meetings; **(D)** operating audio-visual equipment (microphones, projectors, etc.); **(E)** in charge of streaming via webinars, YouTube, etc.; **(F)** troubleshooting in online meetings; **(G)** setting up network; and **(H)** Communicating in English.

The majority (59%, 51/86) responded that their facility did not have a department that supported an international telemedicine program, while the remaining 41% (35/86) responded that they did. All of the TTs indicated that their institution had installed at least one type of VC system (100%, 39/39), and 87% (34/39) indicated that their institution had a conference room equipped with a VC system. More than 90% (36/39) of the TTs had experience in participating in online meetings, and 69% (26/37) had experience organizing online meetings after the COVID-19 outbreak. Most TTs had experience setting up network devices (69%, 27/39) and troubleshooting in online meetings (64%, 25/39). However, only one-third (36%, 14/39) of the participants reported communicating in English (Fig. 1).

### Activities and needs

Eighteen respondents (21%, 18/86) from 14 institutions (33%, 14/42) indicated that they had conducted international programs since the start of the COVID-19 pandemic, whereas 21 respondents (24%, 21/86) from 17 institutions (40%, 17/42) had conducted domestic programs. Regarding the needs of developing new international programs, 19 respondents (22%, 19/86) from 18 institutions (43%, 18/42) indicated that there are needs for international programs, and 20 respondents (23%, 20/86) from 19 institutions (45%, 19/42) indicated needs for domestic programs. However, when examined by the person in charge, about half (41/86) answered “not sure” about the need for information sharing with overseas medical institutions or the need for new programs at their own institutions. Regarding activities, responses of the MI and TT matched in 30% of the facilities (14/47), while in almost half of the facilities (49%, 23/47), the opinions of the respondents differed. Regarding needs, almost half of the facilities (49%, 23/47) matched, while in 34% of the facilities (16/47), the opinions differed.

**FIG. 2.**
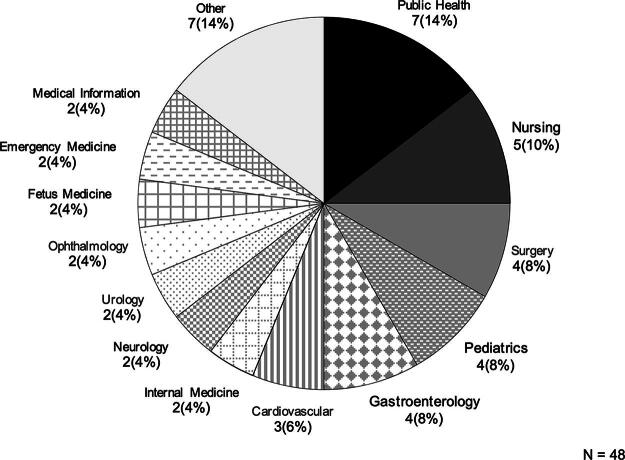
Medical subspecialties for international doctor-to-doctor telemedicine activity.

Based on the responses from the groups that organized international telemedicine conferences/consultations (18 respondents from 14 institutions), public health was the most frequently cited topic (7 respondents), followed by nursing (5), surgery (4), pediatrics (4), and gastroenterology (4; Fig. 2). Of the activities organized, 35% (6/17) involved the sharing of information related to COVID-19. A total of 62 countries were listed as partners of activities. The median number of countries was 3 per institution. At the most, one institution implemented programs with 58 countries. The most frequently mentioned partner countries were Thailand (eight respondents), South Korea (six), and the United States (six). Asia accounted for the largest share (54%, 69/128), followed by Europe (15%, 19/128) and Latin America (9%, 12/128). Developing countries accounted for 70% of the partners (90/128; Table 3). The most common program formats were lectures and seminars for both domestic (18 respondents) and international (14 respondents) events, followed by case consultationss (domestic: 11; international: 9) and live demonstrations (domestic: 7; international: 6; Fig. 3). A comparison of activities in terms of commercial company support showed that 45% (20/44 respondents) of domestic activities received support, while most (88%, 30/34 respondents) of international activities did not, which was a statistically significant difference (*p* < 0.00*; Fig. 4).

**FIG. 3.**
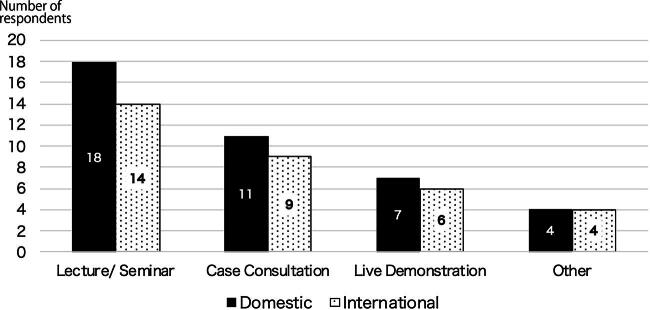
Format of domestic and international doctor-to-doctor telemedicine activity.

**FIG. 4.**
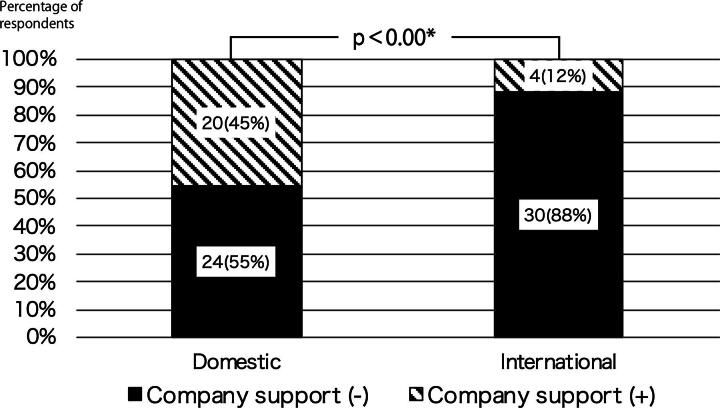
Differences in the number of respondents receiving commercial company support for domestic and international doctor-to-doctor telemedicine activity.

**Table 3.** Partner Countries Involved in International Telemedicine Conferences/Consultations

Region	Number of countries (responses)	Countries (responses)
Asia	26 countries (69 responses)	Thailand (8), Korea (6), Indonesia (5), Myanmar (5), Russia (4), Vietnam (4), Malaysia (4), Philippines (3), Laos (3), China (3), India (3), Pakistan (2), Taiwan (2), Bhutan (2), Bangladesh (2), Cambodia (2), Nepal (2), Kyrgyzstan (1), Turkmenistan (1), Uzbekistan (1), Kazakhstan (1), Singapore (1), Brunei Darussalam (1), Mongolia (1), Hong Kong (1), and Sri Lanka (1)
Europe	14 countries (19 responses)	United Kingdom (4), Sweden (2), Germany (2), Italy (1), Switzerland (1), Norway (1), France (1), Georgia (1), Netherlands (1), Spain (1), Ukraine (1), Austria (1), Azerbaijan (1), and Armenia (1)
Latin America	10 countries (12 responses)	Brazil (3), Mexico (1), Peru (1), Chile (1), Colombia (1), Argentina (1), Nicaragua (1), Paraguay (1), Costa Rica (1), and Dominican Republic (1)
Africa	5 countries (10 responses)	Kenya (3), Egypt (2), Tanzania (2), Nigeria (2), and Angola (1)
North America	2 countries (10 responses)	United States (6) and Canada (4)
Middle East	3 countries (5 responses)	Israel (2), Turkey (2), and United Arab Emirates (1)
Oceania	2 countries (3 responses)	Australia (2) and Fiji (1)

### Barriers

More than half of the respondents (67%, 58/86) reported obstacles in promoting international activities at their institutions, of which 84% (49/58) indicated that the most serious barrier was the lack of human resources at their own institution, including physicians, technical personnel, and coordinators. Only 5% (3/58) of respondents reported technical barriers such as “equipment and network issues” or “shortage of well-equipped meeting rooms” as the most serious barriers to the implementation of international telemedicine conferences/consultations. One MI raised security policy issues as the most serious barrier.

While more groups with no activities (vs. groups with activities) identified the lack of human resources at their own institutions as the greatest barrier (*p* = 0.03, 89%, 42/47 and 64%, 7/11, respectively), there was no difference between groups with and without a support department (*p* = 0.24, 75%, 12/16 and 88%, 37/42, respectively) or MIs/TTs (*p* = 0.05, 93%, 28/30 and 75%, 21/28, respectively).

Of those who answered that there was no support department, 40% (17/42) identified the shortage of doctors as the most serious barrier, whereas only 19% (3/16) identified it among those who answered that they have a support department. Moreover, 13% (2/16) of those who answered that they have a support department identified a shortage of staff at the partner institution, whereas none who answered that they did not have a support department identified this.

**FIG. 5.**
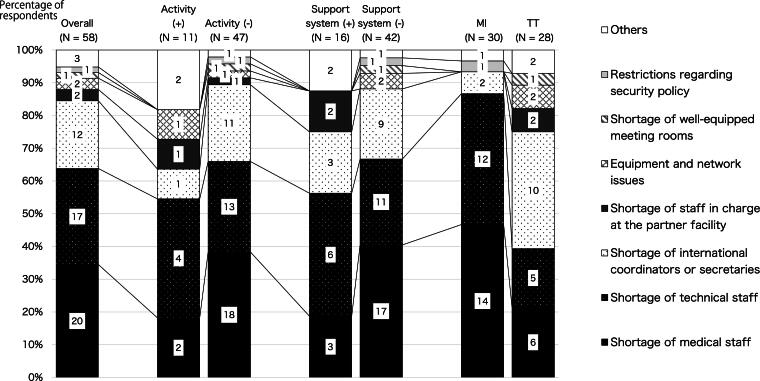
Breakdown of barriers to international doctor-to-doctor telemedicine conferences/consultations by activity status, with/without support department, and staff roles.

Both MIs and TTs identified the lack of human resources as the most serious barrier; but while MIs identified the shortage of MI and TTs (47%, 14/30 and 40%, 12/30, respectively) rather than that of international coordinators or secretaries (7%, 2/30), many TTs (36%, 10/28) identified the lack of coordinators. TTs also identified the shortage of staff at the partner facility and issues in equipment and network, but no MI raised these problems (Fig. 5).

## Discussion

This study revealed the status of international telemedicine conferences/consultations following the COVID-19 pandemic, based on the responses of almost all Japanese national university hospitals (94.2%). Three topics were extracted from this survey: “widespread use of VC and the reduction of technical barriers,” “insufficient human resources to meet the growing needs,” and “improving information sharing and scooping up needs.”

### Principal results

#### Widespread use of VC and the reduction of technical barriers

The penetration rate of VC was 100% (39/39 TTs). This increased compared with the pre-COVID-19 survey (82%).^13^ In addition, while only 72% of the TTs had operated a VC prior to the COVID-19 pandemic, more than 90% of the TTs in the current survey had participated in using a VC on a personal terminal. Nearly 70% had hosted a meeting, and half (49%) had even experienced hosting webinars and live streaming, confirming the widespread use of ICT systems. Of the 58 respondents who reported obstacles in promoting international activities at their institutions, only 5% (3/58) of respondents reported technical barriers such as “equipment and network issues” and “shortage of well-equipped meeting rooms,” indicating that the development and spread of ICT after COVID-19 has reduced the perception of technical hurdles that previously existed in international telemedicine conferences/consultations. Similarly, only one respondent raised the issue of security policy, and this is thought to be closely related to the fact that this study targeted teleconferences/consultations for education and medical treatment support, rather than doctor-to-patient activity. In general, patient information is often not necessary in doctor-to-doctor telemedicine conferences/consultations, and measures are often taken to exclude patient information from communications.^42–44^

However, despite the reduction in technical barriers, lack of technical staff still accounted for the majority of barriers (30%), which may strongly suggest the need for staff to set up software such as Zoom and Webex and prepare video and audio equipment for meeting rooms, so that meetings and workshops can be hosted online following the COVID-19 pandemic.

#### Insufficient human resources to meet the growing needs

Although there was no increase from the previous survey, only about 30% of facilities (13) were very active. Surprisingly, telemedicine conferences/consultations were implemented with 62 countries following the COVID-19 pandemic. Many partner countries are in Asia and developing countries and are likely to base their activities on their own human networks. They held lectures and case consultations both domestically and internationally. In a survey of doctor-to-doctor telemedicine conducted by the Ministry of Internal Affairs and Communications in 2019, covering 64 medical facilities in Japan, the most common response was remote consultations, but even so, only 26% of medical institutions have conducted such activities. This survey did not focus on international activities, and it can be said that the level of activity for international doctor-to-doctor telemedicine conference consultations at Japanese national universities is higher than that at ordinary medical facilities in Japan.^22^ This is likely because university hospitals place more importance on research and education than general hospitals do. At university-affiliated hospitals, international exchange and social contribution activities are often considered in faculty evaluations. However, many hospitals do not include these as evaluation criteria. Public health, which was the most common topic, is globally important from the perspective of universal health coverage. Particularly in early 2020, it was likely necessary for health care workers to share information on infection control, including that pertaining to COVID-19. To support this, the need for international telemedicine conferences/consultations increased more than 1.5 times compared with that in the pre-COVID-19 survey. Before the COVID-19 pandemic, VC was available in most university hospitals; however, its use was limited, and it was a “special technology” in Japanese national university hospitals, requiring technicians for its operation. Therefore, it was probably impractical to use VC to share information with overseas facilities. However, the demand for VC increased after the pandemic, and technological improvements transformed it into a technology that anyone could use. In addition, during the COVID-19 pandemic, there was an increased need to share information on its management methods and operations within hospitals, and training and inspections that had previously been conducted in person needed to be conducted securely over the internet. This has likely led to an increase in the number of I-PT members who feel the need to casually utilize technology for international information sharing using VC. However, the fact that activity has not actually increased means that obstacles have remained, even though the “technical barriers” have been lowered.

Most I-PT members (84%) complained that a lack of human resources was an obstacle to promoting international telemedicine conferences/consultations. Human resources comprised 34% physicians, 29% technical staff, and 21% international coordinators or secretaries, indicating a shortage of all types of staff. Although international telemedicine conferences and consultations are largely voluntary, a diverse range of staff is required. The absence of even one staff type is believed to impose a significant burden on implementation. Furthermore, compared with domestic activities, more international activities were conducted without the support of commercial companies, suggesting that medical device manufacturers and pharmaceutical companies, which primarily market domestically, are less interested in providing financial resources to underwrite international telemedicine conferences/consultations. As communication in English and program coordination are necessary for collaboration with overseas hospitals, the presence of an international coordinator or secretary is important. Currently, I-PT members have defined roles for physicians (MI) and technicians (TT), but no administrative staff has been assigned. In this survey, more TTs (36%) than MIs (7%) raised the lack of international coordinators and secretariats as the biggest barrier. This is probably because many of the TTs do not speak English. Therefore, in the future, measures should be considered for administrative staff who can communicate internationally. It is also expected that administrative tasks will be simplified using learning management systems or dedicated management systems in the case of organizing routine programs.^54–57^ In particular, more members identified the lack of human resources as a barrier at facilities without activities, so it is likely that activities can be increased by taking measures to increase staff. However, there was no significant difference in the presence or absence of support departments or human barriers, so it can be said that to promote activities, human resources that aim to not only support activities but also actively implement the activities themselves are needed. This is similar to the results of researches on international telemedicine that have been carried out to date. For example, a 2008 survey of international support in developing countries found that telemedicine was already providing free and rapid services, but that demand was not growing. The reasons given were “Thatcherism,” “cultural problem of asking for help,” “inappropriate ‘experts,’” “referrers too busy,” and “perceived loss of control.”^58^ Furthermore, a review of international telemedicine conducted in 2012 identified four factors (legal, sustainability, cultural, and contextual) that hinder these activities, with the aim of improving access to specialist services in low- and middle-income countries and rural areas where medical resources are lacking.^21^ This is also related to the fact that the obstacles to international telemedicine in this survey were not technical, likely because some dedicated doctors and departments are working independently along human networks. To continue developing such activities at Japanese national university hospitals, it is necessary to increase the understanding and motivation of medical institutions and departments. It is also proposed that the support system provided by the government and foundations should be expanded, and that international support activities should be incorporated into hospital evaluations.

#### Improving information sharing and scooping up needs

Forty-three percent of the institutions indicated that at least one of the persons in charge needed a new program at their institution. In terms of the person in charge, about half (41/86) were unsure about the need to share information with overseas medical institutions or the need for a new program at their institutions. This survey also revealed differences in perception among the staff at the facilities. The answers of MI and TT differed at around half of the facilities regarding whether activities were being carried out, and at over 30% of the facilities regarding whether there was a need. These results suggest that many I-PT members do not have access to information on hospital-wide international telemedicine conferences/consultations. One reason is that many university hospitals do not have international medical departments, or, if they do, their main focus is on receiving foreign patients, with no organization in charge of international telemedicine programs. Currently, many Japanese universities have international relations (IR) departments to support international exchange, but only 31% (13/42) of national university hospitals had IR departments in 2019.^58^ This is consistent with the results of the current survey, implying that internationalization is still a low priority for national university hospitals. More IR departments must be established to support the expansion of overseas partner activities. Many partner countries are developing countries, including those in Asia, and are likely to base their activities on their own human networks. The continuous exchange of human resources can bring new perspectives and motivations to the field, and financial support is available from humanitarian funds or projects. However, implementing international telemedicine conferences requires creating a human network of connected counterparts. Therefore, it is necessary to encourage health professionals to participate in existing international telemedicine programs, inform them of the benefits of starting and continuing such activities, and gradually change their mindset by creating a human network. For this purpose, a platform for sharing information on existing activities at each university, led by an I-PT group, could be helpful. It would be beneficial to identify the existing activities and encourage participation in them, which in turn will trigger the initiation of these programs in institutions that are not currently engaged.

### Limitations and strengths

This study targeted I-PTs, which were established at Japanese national university hospitals to promote inter-country doctor-to-doctor telemedicine conferences/consultations. However, most respondents said that they spent little time on telemedicine (less than 20% in nearly all cases), and it is difficult to prove that individuals who responded to this survey have complete and reliable knowledge and insight into the organizational barriers, technical issues, and general use of inter-country doctor-to-doctor telemedicine conferences/consultations. Therefore, these results may not reflect the actual telemedicine situations in hospitals. In the future, it may be possible to obtain more practical information by surveying the leaders and managers of each institution. Moreover, this study could not identify the reasons for the low-level needs and activities, or the motivation of enthusiastic staff. It is hoped that these will be identified in the future through qualitative research targeting staff who are either enthusiastic or not enthusiastic about international telemedicine. In addition, this study surveyed the same I-PT as a previous study conducted prior to COVID-19 regarding the use of international telemedicine conferences/consultations. However, the comparisons were limited owing to changes in the question items, which reflected changes in the status of technology utilization before and after the COVID-19 pandemic. However, this national survey is valuable for identifying problems encountered in international telemedicine conferences/consultations following the COVID-19 pandemic. One strength of this study is the very high response rate, with 94% of Japanese university hospitals responding to the survey. Although our survey results are likely to include more negative respondents than a study with a lower response rate, they are robust because the bias caused by a low response rate has been removed.

## Conclusions

We reported the status of international telemedicine conferences/consultations at Japanese national university hospitals following the COVID-19 pandemic based on responses from 94.2% of these hospitals. VC penetration has spread to all universities (100%, 39/39), and most TTs are technically experienced. Therefore, the technical hurdles for utilization are low, and the need for new programs has been growing. However, there is a lack of human resources such as international coordinators and administrative staff to support smooth communication with overseas partners and promote activities on their own.

## Abbreviations Used

COVID-19: The Coronavirus Disease 2019
ICT: Information and Communications Technology
I-PT: Internationalization Project Team
JICA: Japan International Cooperation Agency
MD: Medical Doctor
MI: Medical staff in charge of Internationalization
ODA: Official Development Assistance
TT: Technical staff responsible for Telecommunication
VC: Videoconferencing
